# Supplementary material for: Meta-Analysis of Rice Phosphoproteomics Data to Understand Variation in Cell Signaling Across the Rice Pan-Genome
Source: J Proteome Res. 2024 May 29;23(7):2518–31. doi: 10.1021/acs.jproteome.4c00187 (PMC11232104; doi:10.1021/acs.jproteome.4c00187)
Supplement: Supplementary file 1 — pr4c00187_si_001.pdf [file pr4c00187_si_001.pdf]

Meta-Analysis of Rice Phosphoproteomics Data to Understand Variation in Cell Signaling Across the Rice Pan-Genome

*Kerry A Ramsbottom[1], Ananth Prakash[2], Yasset Perez-Riverol[2], Oscar Martin Camacho[1], Zhi Sun[4], Deepti J. Kundu[2], Emily Bowler-Barnett[2], Maria Martin[2], Jun Fan[2], Dmytro Chebotarov[3], Kenneth L McNally[3], Eric W Deutsch[4], Juan Antonio Vizcaíno[2] and Andrew R Jones[1]**

1. Institute of Systems, Molecular and Integrative Biology, University of Liverpool, Liverpool, L69 7BE, United Kingdom.

2. European Molecular Biology Laboratory, EMBL-European Bioinformatics Institute (EMBL-EBI), Hinxton, Cambridge, CB10 1SD, United Kingdom.

3. International Rice Research Institute, DAPO 7777, Manila 1301, Philippines

4. Institute for Systems Biology, Seattle, Washington 98109, United States.

Corresponding author(s): Andrew Jones (Andrew.Jones@liverpool.ac.uk)

Supplementary Tables and Figures

Supplementary Table 1: Summary of all rice phosphoproteomics datasets identified via the PRIDE repository.

Supplementary Figure 1: Histograms showing scores of potential phosphosites on five peptidoforms identified with pTyr Gold scored sites.

Supplementary Figure 2: Score histograms of peptide modification positions and spectra for a given peptide "IAVQNAETLSLASPR"

Supplementary Figure 3: Spectrum visualisation comparing the potential phosphorylation positions in the peptide IAVQNAETLSLASPR from the “Rice_phos_Nip_0h_20per_F1_R3” experiment file within the dataset PXD004939

Supplementary Figure 4: Significant motifs surrounding the S/T phosphosites for the gold and silver category sites.

Supplementary Figure 5: Significant motifs surrounding the S/T phosphosites for the gold category sites.

Supplementary Table 2: Table showing the presence/absence of significant motifs in each of the category groupings; Gold, Gold and Silver and Gold, Silver and Bronze.

Supplementary Figure 6: Enriched terms for proteins containing each enriched motif across all gold-silver-bronze datasets against all phosphoproteins.

Supplementary Table 1: Summary of all rice phosphoproteomics datasets identified via the PRIDE repository.

PXD	Title	PMID	Instrument	Count of .RAW files	Included in analysis?
PXD000857	29RNP phosphorylation site - Identification and characterization of chloroplast casein kinase II from Oryza sativa (rice)	25316064	LTQ Orbitrap	1	N
PXD000923	Rice Pistil LC-MSMS - Analysis of Phosphoproteome in Rice Pistil	25074045	TripleTOF 5600	10	Y
PXD001168	29RNP kinase - Identification and characterization of chloroplast casein kinase II from Oryza sativa (rice)	25316064	LTQ Orbitrap	6	N
PXD001774	Un-conditioned commercial embryo culture media contain a large variety of non-declared proteins: a comprehensive proteomics analysis	25164020	TripleTOF 5600		N
PXD002222	Rice-xoo interaction phosphoproteome LC-MSMS	26112675	Q Exactive	6	Y
PXD002756	Proteomic and Phosphoproteomic Analyses Reveal Extensive Phosphorylation of Regulatory Proteins in Developing Rice Anthers	26360816	LTQ Orbitrap Elite	5	Y
PXD004705	A phosphoproteomic analysis of ABA induced protein phosphorylation revealed that Serine 71 is a major phosphosite for bZIP72 in SAPK10-mediated ABA signaling	28439285	Q Exactive	9	Y
PXD004939	A comprehensive proteomic survey of ABA-induced protein phosphorylation in rice (Oryza sativa L.)	28054942	Q Exactive	9	Y
PXD005241	A phosphoproteomic landscape of rice (Oryza sativa L.) tissues	28382632	Q Exactive	18	Y
PXD007979	Comparative Qualitative Phosphoproteomics Analysis Identifies Shared Phosphorylation Motifs and Associated Biological Processes in Flowering Plants	29654922	TripleTOF 5600<br>LTQ Orbitrap Velos<br>Q Exactive	124	N

PXD010565	Analysis of chloroplast phosphoproteome	Pre-publication	LTQ Orbitrap	24	N
PXD012764	Phosphoproteomic profiling responds to phosphate starvation in rice	31424513	Q Exactive	6	Y
PXD019291	ABNORMAL POLLEN 1 (AP1) encodes a legume-type lectin receptor-like kinase and is involved in starch accumulation during rice pollen maturation	33658224	Q Exactive	4	Y

Peptide Atlas dataset deposition and visualisation

Localisation probabilities can be viewed through PeptideAtlas (https://peptideatlas.org/builds/rice/phospho/) in the form of histograms. Supplementary Figure 1a shows the histogram of the peptide "IAVQNAETLSLASPR". Here we can see the distribution of scores around each potential phosphorylation site within the peptide. The serine at position 13 shows the best score distribution with 63 PSMs supporting this site within the scores 0.95<p<0.99 and 128 PSMs with scores 0.81<p<0.95. These supporting sites are summarised in Supplementary Figure 1b, with an example spectra for this peptide being displayed in Supplementary Figure 2a for the PSM with the Universal Spectrum Identifier "mzspec:PXD004939:Rice_phos_Nip_0h_20per_F1_R3:scan:23826:IAVQNAETLSLAS[Phospho]PR/2". Supplementary Figure 2B shows how the annotation of the alternative position spectrum changes, highlighting how valuable USIs can be when investigating modification positions.

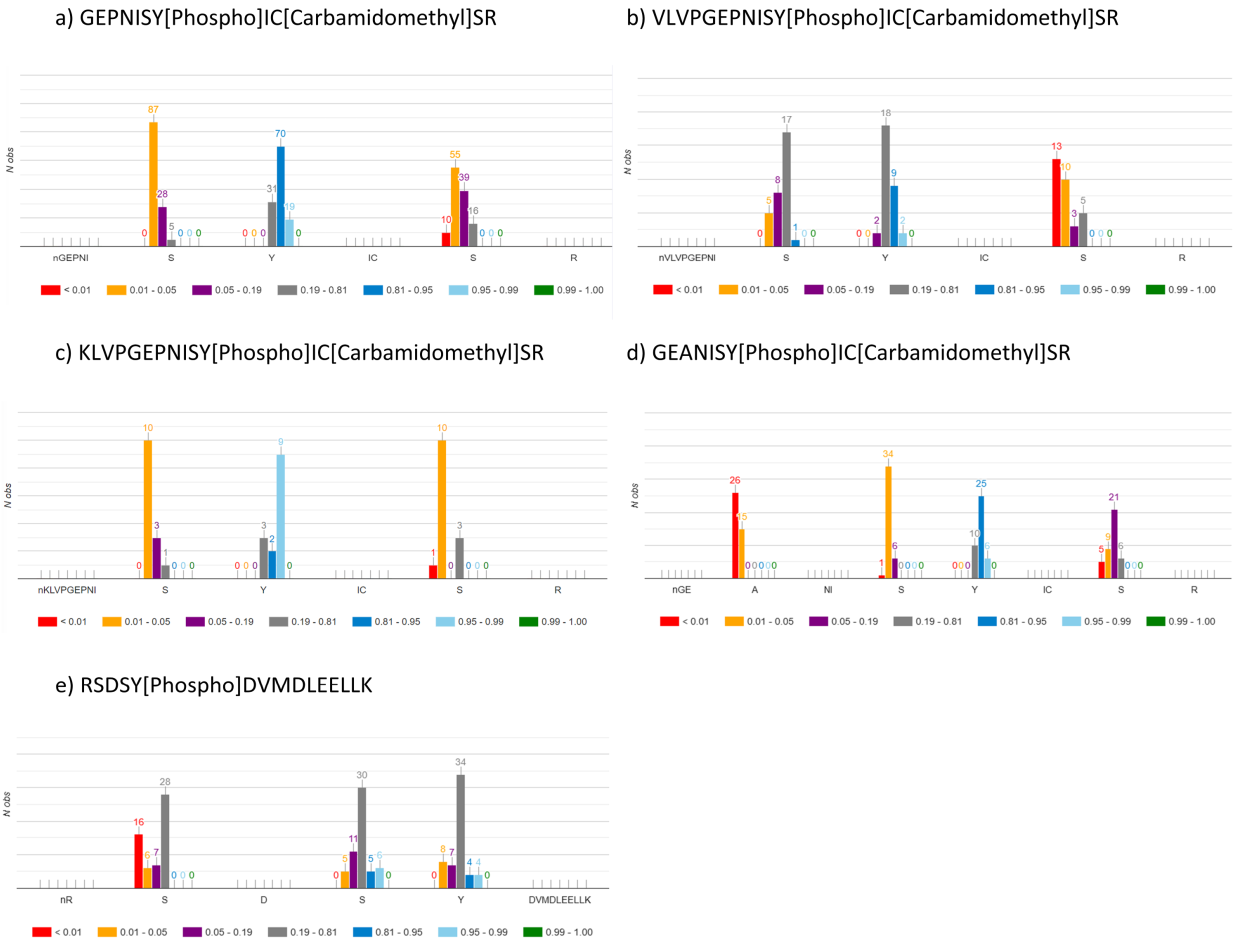

Supplementary Figure 1: Histograms showing scores of potential phosphosites on five peptidoforms identified with pTyr Gold scored sites. a) shows some evidence of phosphorylation of the Tyr site with 70 spectra falling within the "0.81-0.95" and 19 spectra in the "0.95-0.99" high confidence categories, showing that this could potentially be a true

identification , b) shows some weak evidence for the Tyr site with 9 spectra in the “0.81-0.95” and 2 spectra in the “0.95-0.99” categories, c) shows some weak evidence for the Tyr site with 9 spectra being seen within the “0.95-0.99” score bin, d) shows some fairly strong evidence of the Tyr site with 25 spectra in the “0.81-0.95” and 6 within the “0.95-0.99” high confidence score categories and e) shows weak evidence for the Y with 4 spectra within the “0.81-0.95” and 4 spectra in the “0.95-0.99” score ranges, with similar evidence being seen for the Ser at position 4, with 5 spectra within the “0.81-0.95” and 6 spectra in the “0.95-0.99” score ranges . Refer to Supplementary Figure 2 for more detail on Peptide Atlas build.

a)

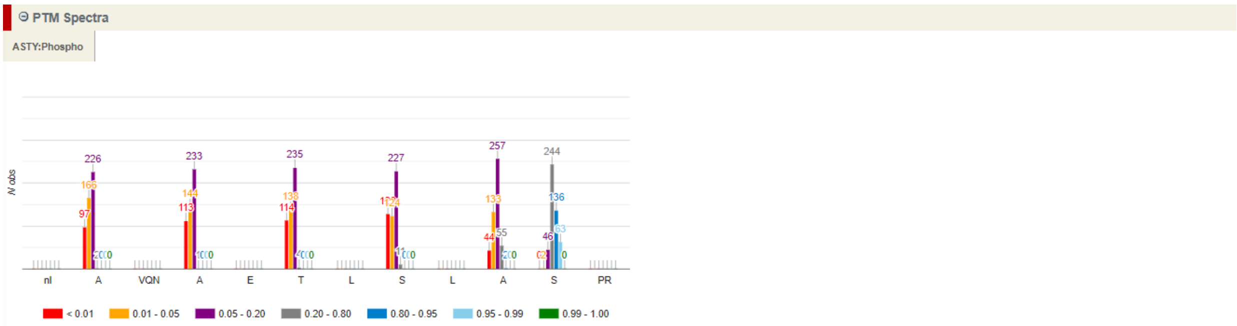

b)

Download as: TSV

Modified Sequence	PTM Sequence	Charge	Expt	Instr	Prob	Spectrum Name	Fragmentation Type	Spectrum	Precursor Intensity (x10^6)	TIC (x10^6)	S/N	PTM Lability
IAVQNAETLSLAS[Phospho]PR	I AVQNAETLSLASPR	3	9,196	Q Exactive Plus	0.998	Rice_leaf_0h_phospho_test2.20652.20652.3	HR HCD		n/a	2.01	n/a	0.03484
IAVQNAETLSLAS[Phospho]PR	I AVQNAETLSLASPR	3	9,197	Q Exactive Plus	1	Rice_phos_ABA_12h_20per_F1_R2.23916.23916.3	HR HCD		n/a	3.20	n/a	0.01902
IAVQNAETLSLAS[Phospho]PR	I AVQNAETLSLASPR	3	9,195	Q Exactive Plus	1	Rice_phos_BR_12h_20per_F1_R1.21445.21445.3	HR HCD		n/a	1.38	n/a	0.02903
IAVQNAETLSLAS[Phospho]PR	I AVQNAETLSLASPR	3	9,195	Q Exactive Plus	1	Rice_phos_BR_3h_20per_F1_R2.23743.23743.3	HR HCD		n/a	2.11	n/a	0.02041
IAVQNAETLSLAS[Phospho]PR	I AVQNAETLSLASPR	3	9,195	Q Exactive Plus	1	Rice_phos_Nip_0h_20per_F1_R1.24070.24070.3	HR HCD		n/a	2.88	n/a	0.09746
IAVQNAETLSLAS[Phospho]PR	I AVQNAETLSLASPR	3	9,197	Q Exactive Plus	1	Rice_phos_Nip_0h_20per_F1_R1.24070.24070.3	HR HCD		n/a	2.88	n/a	0.09656
IAVQNAETLSLAS[Phospho]PR	I AVQNAETLSLASPR	3	9,197	Q Exactive Plus	1	Rice_phos_ABA_12h_20per_F1_R3.23968.23968.3	HR HCD		n/a	3.19	n/a	0.05369
IAVQNAETLSLAS[Phospho]PR	I AVQNAETLSLASPR	3	9,197	Q Exactive Plus	1	Rice_phos_Nip_0h_20per_F1_R3.23901.23901.3	HR HCD		n/a	3.52	n/a	0.1203

Supplementary Figure 2: a) Score histograms of peptide modification positions for peptide “IAVQNAETLSLASPR”, showing favouring of the serine at position 13 with more confident score distributions seen at this position than other potential sites. b) Summary table showing spectra supporting the peptide shown in a).

a)

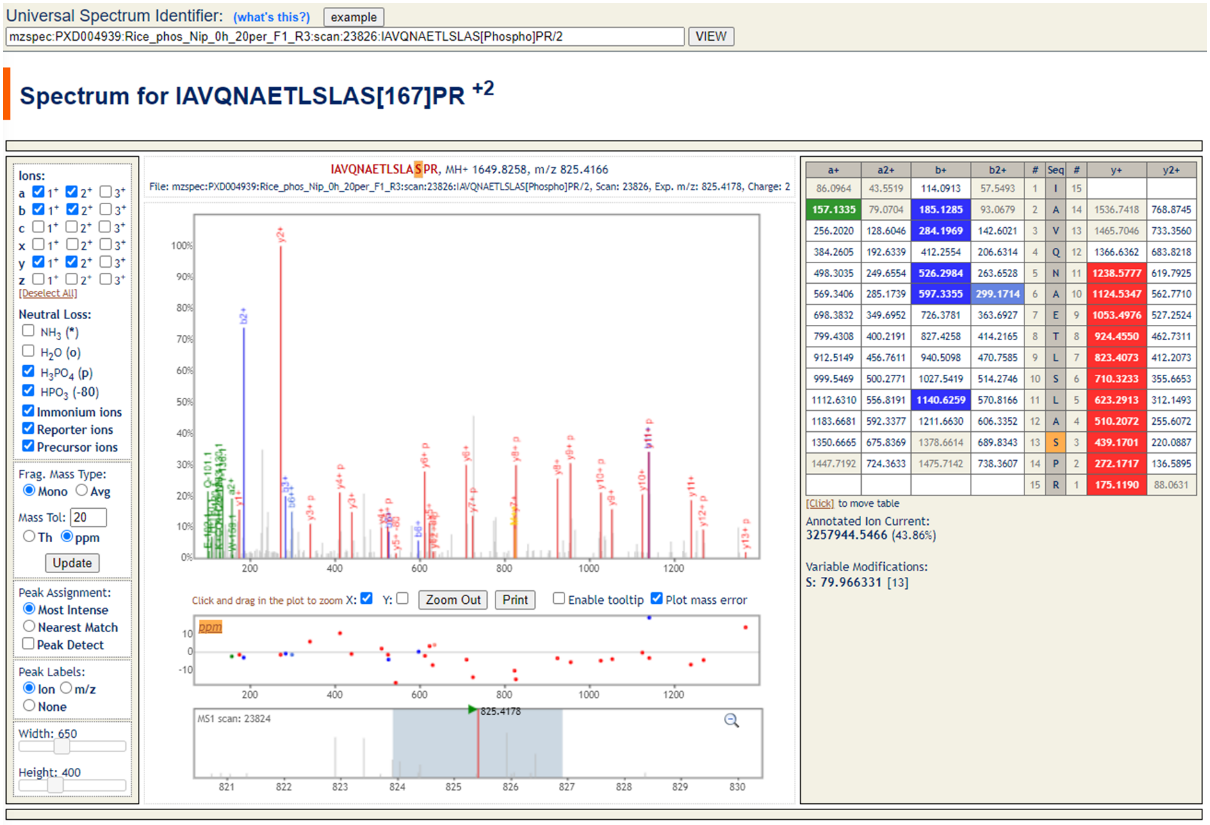

b)

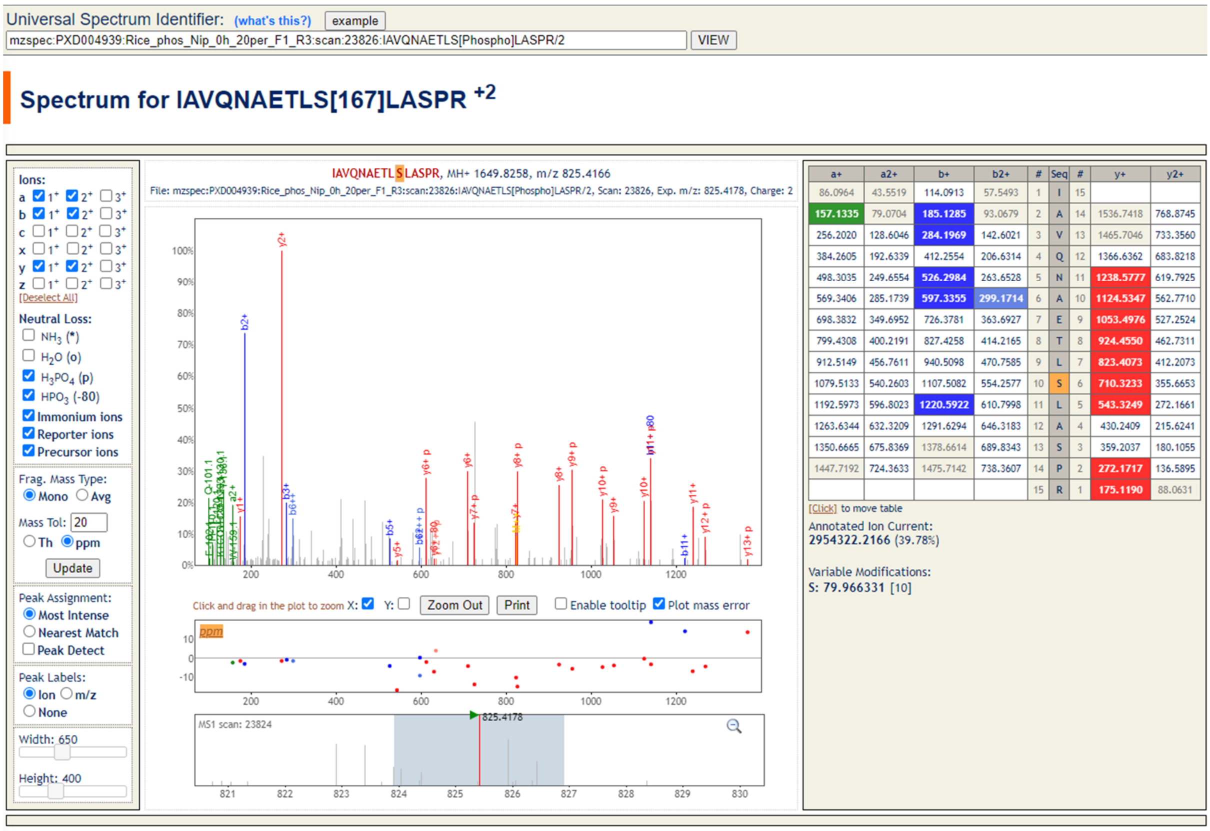

Supplementary Figure 3: Spectrum visualisation comparing the potential phosphorylation positions in the peptide IAVQNAETLSLASPR from the "Rice_phos_Nip_0h_20per_F1_R3" experiment file within the dataset PXD004939. a) shows the spectra with the modification on position 13 (IAVQNAETLSLAS[Phospho]PR), with excellent y-ion coverage supporting the

pS site. b) shows the same peptide with modification at position 10 (IAVQNAETLS[Phospho]LASPR), here the spectra has less coverage being seen in the y-ions.

Motif analysis

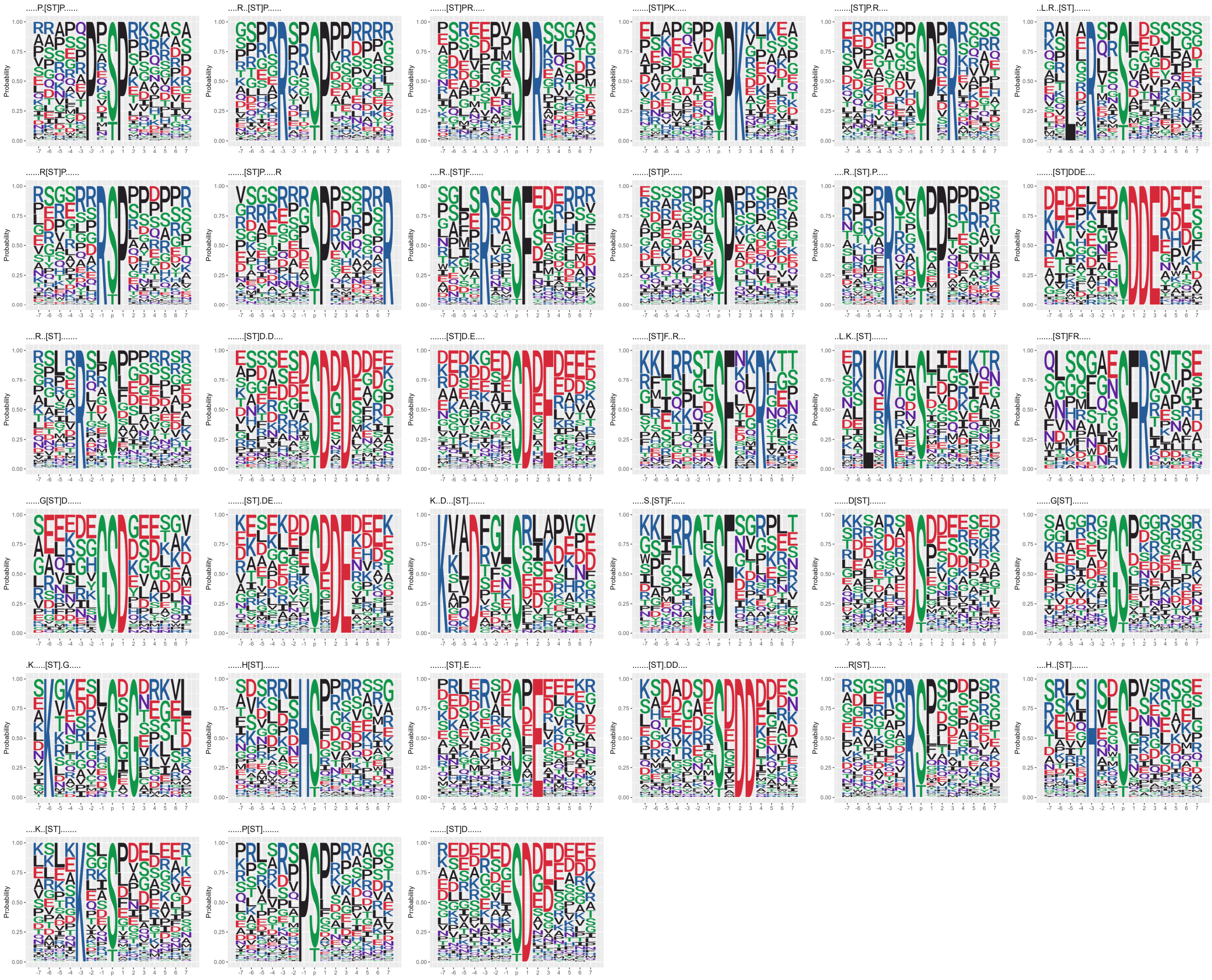

Supplementary Figure 4: Significant motifs surrounding the S/T phosphosites for the gold and silver category sites.

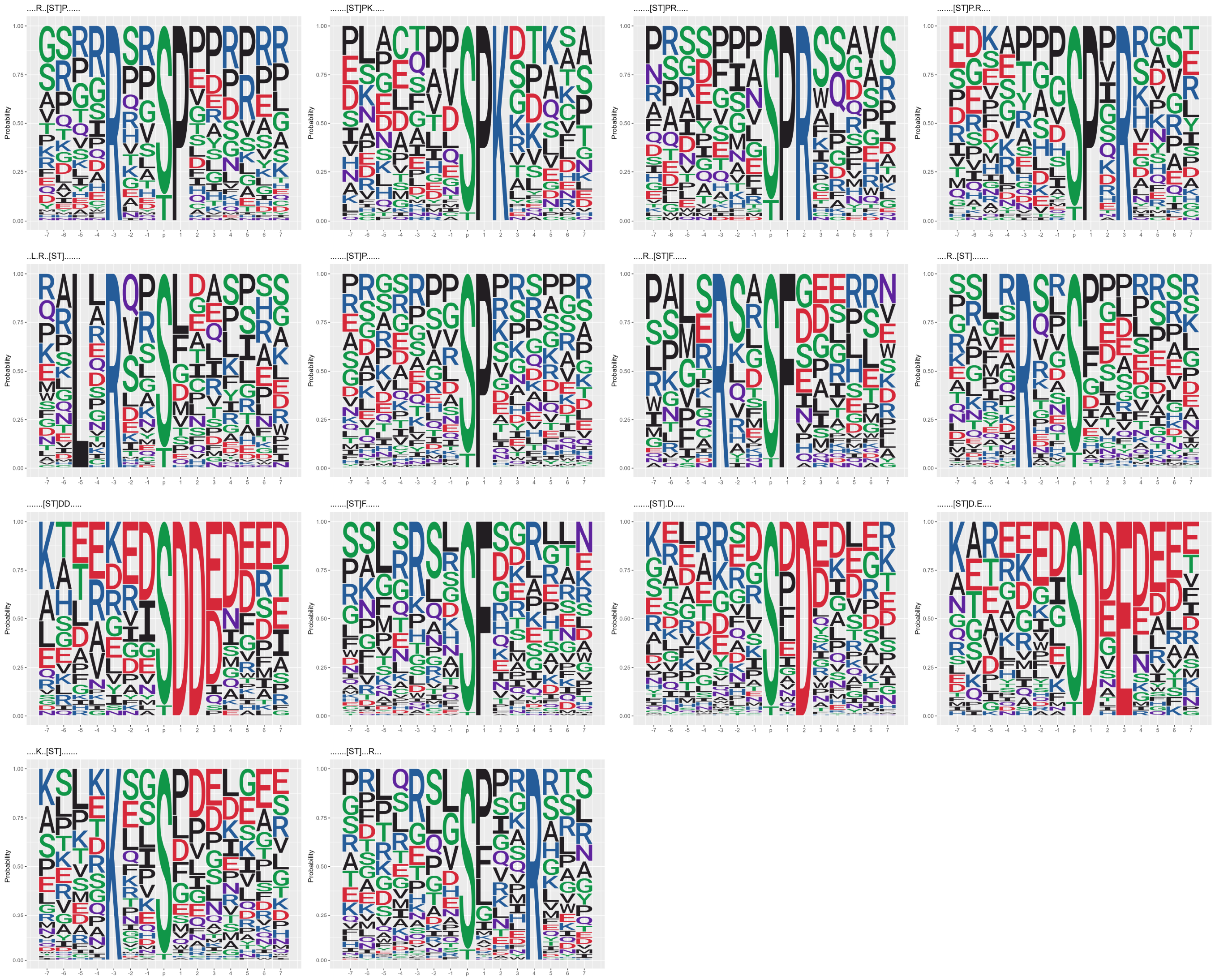

Supplementary Figure 5: Significant motifs surrounding the S/T phosphosites for the gold category sites.

Supplementary Table 2: Table showing the presence/absence of significant motifs in each of the category groupings; Gold, Gold and Silver and Gold, Silver and Bronze.

Motif	Gold	Gold-Silver	Gold-Silver-Bronze
....R..[ST]P......	1	1	1
.......[ST]PK.....	1	1	1
.......[ST]PR.....	1	1	1
.......[ST]P.R....	1	1	1
..L.R..[ST].......	1	1	1
.......[ST]P......	1	1	1
....R..[ST]F......	1	1	1
....R..[ST].......	1	1	1

.......[ST]DD.....	1	0	0
.......[ST]F......	1	0	1
.......[ST].D.....	1	0	1
.......[ST]D.E....	1	1	1
....K..[ST].......	1	1	0
.......[ST]...R...	1	0	0
.....P.[ST]P......	0	1	1
......R[ST]P......	0	1	1
.......[ST]P.....R	0	1	0
....R..[ST].P.....	0	1	0
.......[ST]DDE....	0	1	1
.......[ST]D.D....	0	1	1
.......[ST]F..R...	0	1	1
..L.K..[ST].......	0	1	1
.......[ST]FR.....	0	1	1
......G[ST]D......	0	1	0
.......[ST].DE....	0	1	1
K..D...[ST].......	0	1	0
.....S.[ST]F......	0	1	1
......D[ST].......	0	1	1
......G[ST].......	0	1	1
.K.....[ST].G.....	0	1	0
......H[ST].......	0	1	1
.......[ST].E.....	0	1	1
.......[ST].DD....	0	1	0
......R[ST].......	0	1	0
....H..[ST].......	0	1	0
......P[ST].......	0	1	0
.......[ST]D......	0	1	0
.....P.[ST]P.K....	0	0	1
....RS.[ST]F......	0	0	1
......G[ST]P......	0	0	1
....RS.[ST].......	0	0	1
.......[ST]P...K..	0	0	1
....R..[ST].D.....	0	0	1
......G[ST]...R...	0	0	1
.......[ST]DDD....	0	0	1
......D[ST]D......	0	0	1
.......[ST].D....K	0	0	1
....H.D[ST].......	0	0	1
.K.N...[ST].......	0	0	1
.......[ST]SP.....	0	0	1
.......[ST]E.E....	0	0	1
K....G.[ST].......	0	0	1
....HS.[ST].......	0	0	1
.....R.[ST]..P....	0	0	1
.......[ST].G.....	0	0	1

......D[ST].E.....	0	0	1
.....S.[ST].......	0	0	1
....HV.[ST].......	0	0	1

Pathway enrichment analysis

Pathway enrichment analysis was used to identify pathways involved in proteins containing significant motifs identified, against all rice phosphoproteins, using the ClusterProfiler R package. The proteins containing each of the identified enriched motifs, across all categories (Gold, Silver and Bronze), were searched against a background of all phosphoproteins. “RNA binding” is seen commonly enriched across multiple motifs, as is “nuclear speck” and “mRNA splicing, via spliceosome”. Supplementary Figure 6 shows the dotplots of enrichment terms per motif.

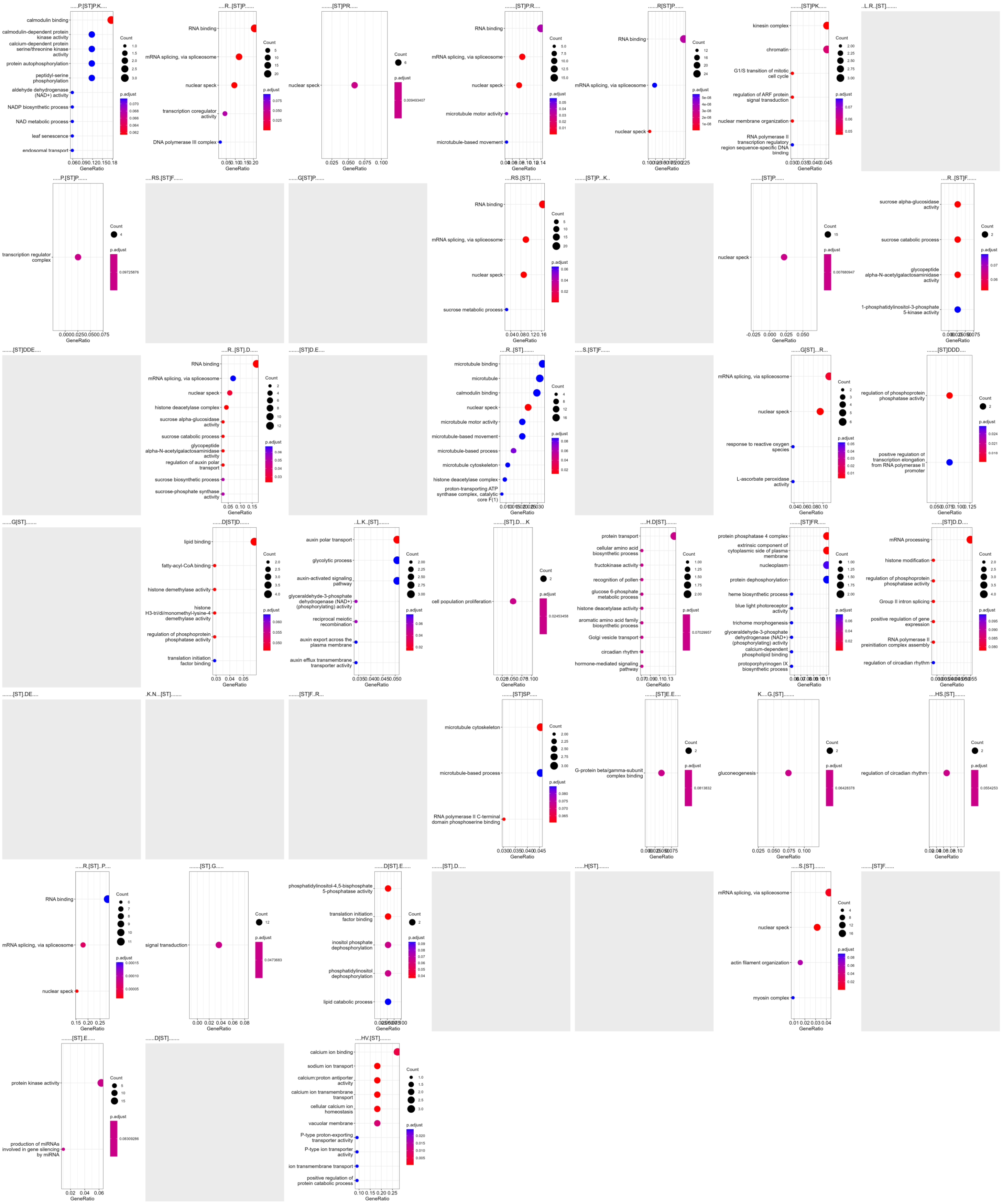

Supplementary Figure 6: Enriched terms for proteins containing each enriched motif across all gold-silver-bronze datasets against all phosphoproteins. Blank plots show motifs which contained no significant motifs.
